# Rhythms of Consciousness: Binocular Rivalry Reveals Large-Scale Oscillatory Network Dynamics Mediating Visual Perception

**DOI:** 10.1371/journal.pone.0006142

**Published:** 2009-07-03

**Authors:** Sam M. Doesburg, Jessica J. Green, John J. McDonald, Lawrence M. Ward

**Affiliations:** 1 Psychophysics and Cognitive Neuroscience Laboratory, Department of Psychology, University of British Columbia, Vancouver, British Columbia, Canada; 2 Department of Psychology, Simon Fraser University, Burnaby, British Columbia, Canada; 3 Brain Research Centre, University of British Columbia, Vancouver, British Columbia, Canada; National Microelectronics Center, Spain

## Abstract

Consciousness has been proposed to emerge from functionally integrated large-scale ensembles of gamma-synchronous neural populations that form and dissolve at a frequency in the theta band. We propose that discrete moments of perceptual experience are implemented by transient gamma-band synchronization of relevant cortical regions, and that disintegration and reintegration of these assemblies is time-locked to ongoing theta oscillations. In support of this hypothesis we provide evidence that (1) perceptual switching during binocular rivalry is time-locked to gamma-band synchronizations which recur at a theta rate, indicating that the onset of new conscious percepts coincides with the emergence of a new gamma-synchronous assembly that is locked to an ongoing theta rhythm; (2) localization of the generators of these gamma rhythms reveals recurrent prefrontal and parietal sources; (3) theta modulation of gamma-band synchronization is observed between and within the activated brain regions. These results suggest that ongoing theta-modulated-gamma mechanisms periodically reintegrate a large-scale prefrontal-parietal network critical for perceptual experience. Moreover, activation and network inclusion of inferior temporal cortex and motor cortex uniquely occurs on the cycle immediately preceding responses signaling perceptual switching. This suggests that the essential prefrontal-parietal oscillatory network is expanded to include additional cortical regions relevant to tasks and perceptions furnishing consciousness at that moment, in this case image processing and response initiation, and that these activations occur within a time frame consistent with the notion that conscious processes directly affect behaviour.

## Introduction

Consciousness has been envisioned as a dynamic global workspace wherein unified experience is assembled out of relevant constituent elements [1], [2]. This view is consistent with the notion that consciousness is chiefly characterized by the qualities of dynamism, selectivity, and integrated subjective experience, attributes explained by the postulation that experience is equivalent to informational integration of relevant neural elements into a large-scale complex [3]. In such a view, the contents of experience would be defined by the activity of the largest and most dominant coalition of functionally integrated neurons at a given moment [3], [4]. Any such process would necessitate continuous and complex rearrangement of neural populations across widespread and diverse cortical regions, a feat that has been attributed to oscillatory dynamics [e.g. 5]. Low gamma-band (30 Hz to 50 Hz) synchronization between neural groups coding the various features of objects currently populating experience has been proposed as a mechanism for such dynamic functional integration in the brain, and has been suggested to be the biological basis of perceptual experience and feature binding [6]–[8]. It has been proposed that synchronization enables transient functional integration between specific neural groups as bursts of action potentials are consistently exchanged during the depolarized phase of the receiving neurons' ongoing membrane potential fluctuations, thereby enhancing communication between populations oscillating in synchrony [9]. Support for this notion can be drawn from findings that mutual influence between neural populations is positively correlated with gamma-band synchronization in both intra-regional and large-scale oscillatory dynamics [10], [11].

Empirical evidence for the involvement of gamma-band neural synchronization in perceptual binding and awareness flows from diverse lines of research. Gamma-band synchronization in primary visual cortex of cats, for example, occurs most strongly between columns responding to a common object, presumably implementing feature binding and figure-ground segregation [12]–[14]. Stimulus dependent synchronization in the gamma frequency range has also been recorded between cortical areas [15]–[17]. Local and long-range gamma-band electroencephalographic (EEG) phase synchronization have been shown to index the onset of coherent visual perception [18], [19]. The integration of local features in one visual hemifield into a global percept involving both hemifields, as well as the perception of apparent motion across visual hemifields, is accompanied by interhemispheric gamma-band EEG phase coupling [20], [21]. Results such as these have led to the postulation that the emergence of organized perception corresponds to the reordering of a large-scale representational ensemble by way of oscillatory synchronization in the gamma frequency range [22]. We propose that this mechanism can account for the selective integration of contents into a large-scale neural coalition determining the momentary furnishings of experience. The parameters governing cortical oscillations are also dynamic, allowing for the integration, disintegration, and reconstruction of gamma-oscillatory assemblies in time. Accordingly, we propose that large-scale gamma-band synchronization constitutes an oscillatory substrate for the stream of consciousness.

Evidence for this proposed essential relationship between gamma oscillations and consciousness is not limited to feature binding. Large-scale gamma EEG synchronization is greater when a masked stimulus is consciously perceived than when it is not [23]. Similarly, rapid serial visual presentation has been used to demonstrate that conscious perception of a stimulus is associated with greater inter-regional gamma-band phase synchronization, relative to stimuli that are not perceived [24]. The onset of a new visual percept, gauged by perceptual switching in binocular rivalry, coincides with a transient increase in inter-regional gamma-band phase synchronization [25]. Cognitive processes closely associated with consciousness, namely attention and working memory, have also been robustly associated with the synchronization of gamma rhythms [see 26 for review]. Previous research has also revealed that coherent thalamo-cortical and cortico-cortical gamma rhythms are associated with mammalian consciousness, as they are characteristic of CNS states associated with experience (wakefulness and REM sleep). Such rhythms also have been proposed to be the neurobiological basis for consciousness, a view supported by the observation that functional decoupling of thalamus and cortex is a cardinal property of general anesthesia [8], [27]–[30].

If gamma rhythms embody an essential feature of the biological basis of consciousness, and if gamma-band synchronization of selective neural populations constructs a large-scale complex defining the contents of consciousness, as we have proposed, then some mechanism must exist to govern the evolution of such a network over time in order to account for the dynamism of experience. Notably, modulation of intra-regional gamma-band (80–150 Hz) synchronization at a frequency in the theta band (4–7 Hz) has been observed using subdural electrodes on human cortex, with gamma envelope amplitude being most pronounced during the trough of the theta cycle [31]. Moreover, this relationship is accentuated during active cognitive processing, particularly within task-relevant cortical regions [31]. This important result suggests that the construction and degradation of low gamma-band (30–50 Hz) oscillatory neural ensembles might also be governed by theta rhythms. Locking of low gamma-band synchronization to theta oscillations has been associated with an array of cognitive processes including working memory, attention, and perceptual organization [19], [25], [32]–[34]. Transient and periodic desynchronization of gamma rhythms, or phase scattering, has also been observed between periods of synchronization [i.e. 19]. Similarly, periods of recurrent increased long-range gamma-band phase locking consistent with a theta-frequency modulation are often interposed with periods of baseline level phase locking [25], [35]. We interpret such results as indications that theta-modulated gamma synchronization serves to organize transient functional networks across time. Specifically, we propose that large-scale ensembles synchronously oscillating in the low gamma frequency range enable transient functional integration of task- and/or percept-specific neural populations, and that theta rhythms govern the temporal dynamics according to which the life cycle of individual gamma-oscillatory ensembles are organized. This interpretation also suggests that only one truly discrete perceptual experience may exist within a single theta cycle, and that the emergence of new perceptual experiences may be time locked to a particular phase of ongoing cortical theta rhythms.

Ascertaining the neural correlates of perceptual consciousness requires separation of brain processes related to experience from those embodying non-conscious stimulus processing, a distinction made possible using binocular rivalry [36]. Binocular rivalry has been employed to study the relationship between neural oscillations and consciousness using flickering, frequency-tagged stimuli to identify neural populations responding to rivaling images. Such studies revealed that when a frequency-tagged image is perceived, increased local and long-range neural synchronization is observed at the flicker frequency of the stimulus, reinforcing the view that consciousness is associated with large-scale synchronously oscillating neural ensembles [37], [38]. Localization of magnetoencephalographic rhythms generated in this fashion reveals widely distributed, localized sources of flicker-entrained neural activity in the cortex [39]. Importantly, when the flickering stimulus is dominant in the rivalry, and is thus perceived consciously, inter-regional phase synchronization between the locally synchronous sources at the tagged frequency is also at its peak, and decreases sharply when the flickering stimulus becomes suppressed and disappears from consciousness. This incisive study indicates that the emergence of a new visual percept relates to the synchronization of rhythms within distributed cortical regions and the formation of a large-scale oscillatory network by means of inter-regional synchronization, consistent with the notion that visual consciousness is embodied by large-scale coalitions of neurons wherein the contents of perception correspond to what is represented by the victor among competing ensembles [4]. This is the current horizon of our understanding. Unfortunately, because these seminal studies focused on *exogenously* induced rhythms, this understanding leaves undetermined which *endogenous* rhythms might govern dynamic cortical networks relevant to conscious perception.

We propose that the contents of consciousness are defined by which neural populations are integrated into a large-scale gamma-synchronous ensemble at any given time. We further propose that the formation and dissolution of these functional assemblies occurs at a frequency in the theta band, which effectively places temporal constraints on the emergence of new, discrete, perceptual experiences. Two previous key results support this view: (1) When a stimulus is perceived, a second stimulus occurring approximately 180 ms to 500 ms afterwards often fails to reach consciousness [40]. This time range corresponds to oscillation frequencies from about 5.5 Hz to 2 Hz, overlapping the lower part of the classical theta band (4–7 Hz). Moreover, performance on the second stimulus is worst in most experiments at an interstimulus interval of about 225 ms, which corresponds to about 4.4 Hz, a low-theta frequency. We interpret such results as indications that one can have only one discrete experience every theta cycle, effectively implementing a ‘speed limit’ on conscious perception. This phenomenon, dubbed the “attentional blink” has previously been proposed to originate at least partly from the suppression of gamma rhythms, and it has been demonstrated that increased inter-regional gamma-band EEG phase synchronization is associated with successful target detection under these rapid serial visual presentation conditions [24], [41]. (2) In a previous study we demonstrated that the onset of a new percept in binocular rivalry coincides with a burst of large-scale gamma-band phase synchronization which was situated within a procession of gamma-band synchronizations that periodically recurred at a theta rate [25]. These previous findings have led us to the hypothesis that the onset of new visual percepts may be implemented by a recurrent gamma-oscillatory network that is phase-locked to an ongoing cortical theta rhythm.

**Figure 1:**
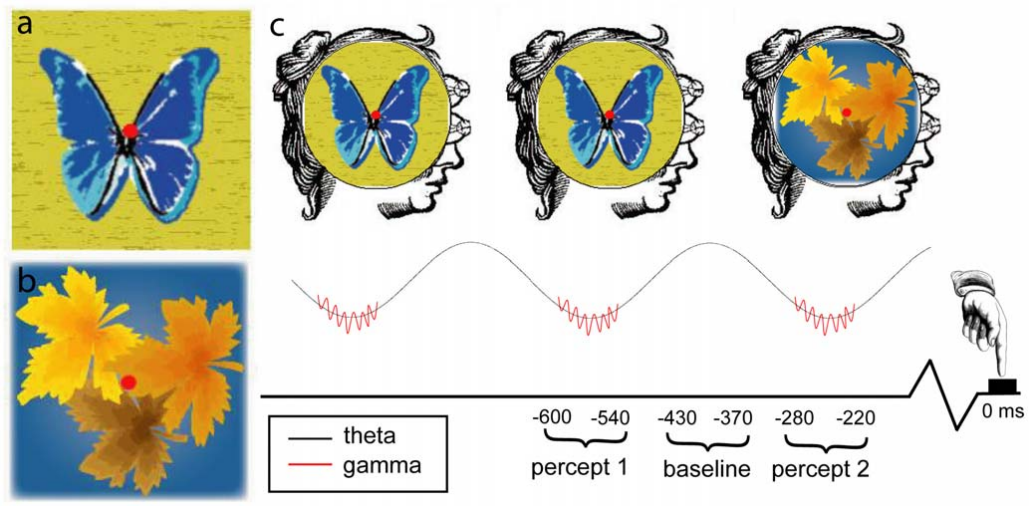
a,b) The left and right eye stimuli, respectively. c) Schematic representation of the stream of perceptual consciousness wherein discrete moments of perceptual experience coincide with gamma-band synchronization, itself locked to a theta cycle. Periodic gamma-band synchronization is locked to the onset of new conscious percepts and hence to button presses signaling perceptual switching. We imaged these oscillatory cortical networks by comparing gamma synchronization during periodic activations to the intervening period of relative desynchronization. In this figure 0 ms indicates button presses indicating the onset of a new percept. The preceding −600 to −540 ms and −280 to 220 ms analysis windows, as well as the −430 to −370 ms baseline interval, are depicted.

In order to test the hypothesis that large-scale gamma-oscillatory neural assemblies of relevant cortical regions are synchronized and desynchronized according to a theta cycle that also determines the timing of new perceptual experiences, we recorded EEG data while subjects viewed rivaling visual images and pressed buttons to indicate which stimulus was being perceived at any given moment. Data epochs time-locked to button presses indicating perceptual switching were extracted from the continuous recording. The resulting time series of scalp voltages were then transformed into the frequency domain to reveal periodic gamma activations that recur at a theta rate preceding the button presses indicating the onset of new percepts. Beamformer analysis was employed to reveal the cortical generators of transient and periodic gamma-band activations (local synchronizations) locked to a theta cycle by comparing these periods to the interposed period of relative inactivity (local desynchronization) between them (Figure 1). A dipolar source montage was seeded according to foci of cortical gamma-band activations identified by beamformer and filtered time series from these sources were assessed for cross-frequency coupling between theta phase and gamma amplitude. Inter-regional gamma-band phase synchronization was also assessed between cortical generators of gamma rhythms during these periods of activity, as was cross-frequency coupling between theta phase in the relevant generators of a pair and gamma-band phase synchronization between them. These analyses directly test the hypothesis that theta-modulated gamma-band synchronization within a network of relevant cortical regions represents the periodic formation of transient functional ensembles relevant to perceptual experience.

## Results

**Figure 2:**
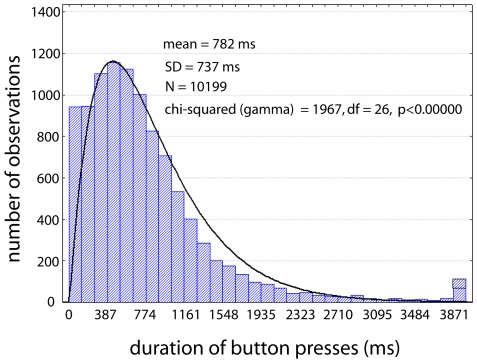
Distribution of durations for perceptual dominance periods. Data shown here were obtained from the 9 subjects included in the EEG analyses. Black curve indicates prediction of a gamma distribution with scale parameter = 400, shape parameter = 2.1, fitted to durations<4001 ms. Although the chi-squared value is highly significant the distribution fits well. It deviates most markedly for the shortest durations (of which there are more than predicted) and durations from about 1200 ms to 2000 ms (of which there are fewer than predicted).

Binocular rivalry induced stable patterns of perceptual dominance with a mean duration of 782 ms and a standard deviation of 737 ms (Figure 2). The frequency distribution of dominance periods was well-fitted by a gamma distribution in line with previous binocular rivalry results [42], although the highly significant chi-squared value indicates that there are some departures.

### Periodic gamma-band activation time-locked to perceptual switching

**Figure 3:**
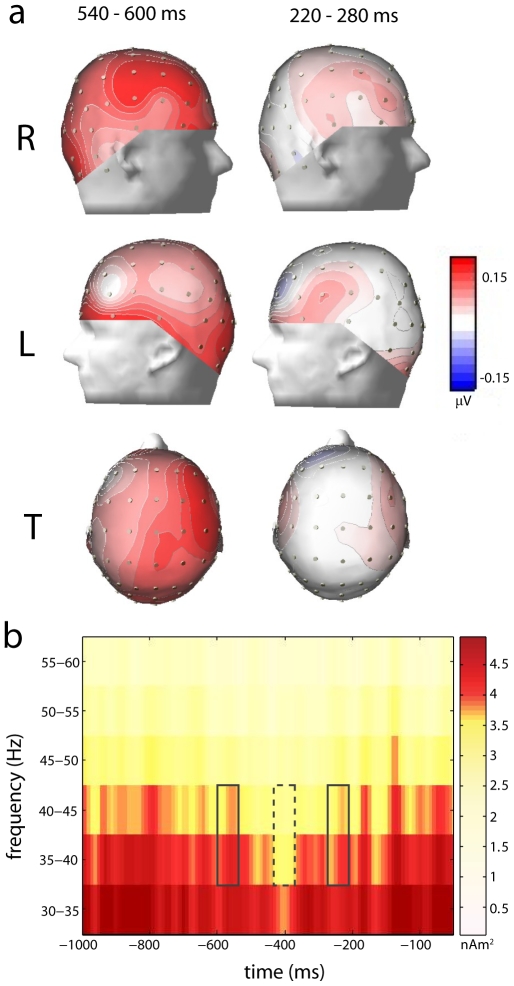
a) Topography of 35–45 Hz scalp spectral power during the −540 to −600 ms and −220 to −280 ms intervals, relative to the baseline interval, for the (R) right side, (L) left side and (T) top view. b) Periodic bursts of gamma-band scalp activity time-locked to button presses (at 0 ms) indicating perceptual switching. Depicted is gamma-band power averaged across subjects and across the 30 electrodes where gamma activity was most clearly expressed (see Methods). Solid lines denote time-frequency windows used for beamformer source localization; dotted lines denote the baseline.

Transformation of EEG scalp activity into the frequency domain, performed using the Brain Electrical Source Analysis software suite (BESA 5.2; Megis Software), revealed transient and periodic gamma activations preceding button presses indicating the onset of a new percept. These transient increases in local gamma synchronization displayed a frontocentral scalp distribution (Figure 3a) and recurred at a rate consistent with a theta cycle, as well as displaying a trough at about 400 ms prior to button presses (Figure 3b).

Beamformer analysis was employed to image the neural generators of gamma-band activation occurring 220–280 ms and 540–600 ms prior to responses, relative to a window of equivalent length in the intervening period from 370–430 ms prior to responses (see Figure 3b). This baseline period interposed between two windows of interest was chosen because gamma-band phase-scattering, which occurs between periods of gamma synchronization, is understood to be a period wherein transient oscillatory networks are dissolved (see discussion). Accordingly, this analysis aimed to image a recurrently activated coalition of cortical regions relative to a period during which this network would be desynchronized (see Figure 1), i.e. during the trough in gamma spectral power at around 400 ms prior to a response. The −220 to −280 ms and −540 to −600 ms intervals were chosen because (i) these were distinct peaks in the recurrent gamma activation relative to the intervening baseline period, (ii) they are consistent with earlier results pertaining to gamma-band neural synchronization time-locked to perceptual switching in binocular rivalry obtained using a similar paradigm [25], and (iii) the −220 to −280 ms period corresponds to reaction times expected in a simple perceptual task, suggesting that perceptual switching during this period may have initiated behavioural responses.

Beamformer analysis imposes a spatial filter to identify activation within a specified time-frequency window. For each voxel, an activation value is assigned by removing correlations with all other voxels. A frequency window of 35 Hz to 45 Hz was chosen for beamformer source analysis as this bandwidth has been most reliably associated with both intra-regional and inter-regional gamma-band synchronization relevant to conscious experience, feature binding, the dynamics of perceptual organization, and specifically perceptual switching in binocular rivalry [12]–[21], [25]–[28].

### Distributed gamma-oscillatory networks time-locked to perceptual switching

**Figure 4:**
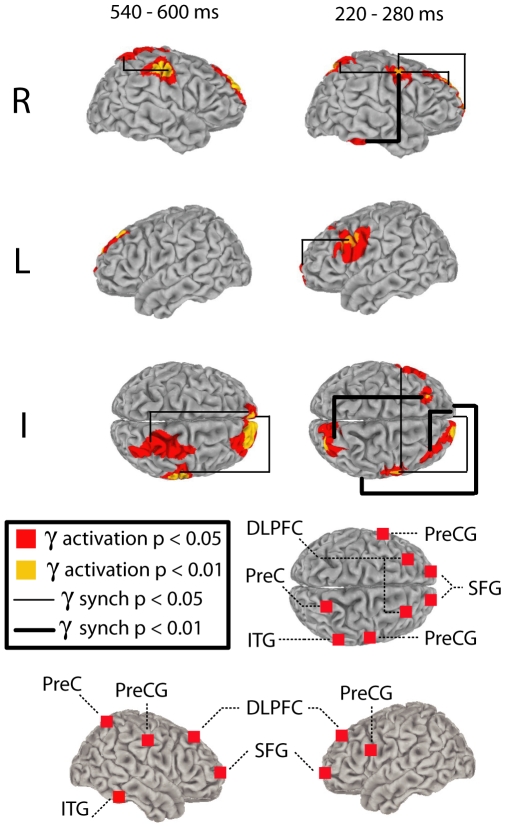
Surface projected regional gamma-band activations, and inter-regional gamma-band synchronization in the 540–600 ms and 220–280 ms pre-response intervals. For clarity, separate images are provided for (R) right intrahemispheric, (L) left intrahemispheric and (I) interhemispheric synchronization across cortical regions. Lower brain figures show surface projected anatomical loci for all identified gamma-band activations.

Beamformer imaging of gamma-band activation revealed multiple generators in both 220–280 ms and 540–600 ms pre-response windows. In the 540–600 ms pre-response interval, activation reached the 0.05 level of statistical significance in right precuneus (PreC), bilateral dorsolateral prefrontal cortex (DLPFC), bilateral superior frontal gyrus (SFG) and right precentral gyrus (PreCG); in the −220 to −280 ms interval all aforementioned sources were significantly activated as well as left precentral gyrus and right inferior temporal gyrus (ITG) (Table 1; Figure 4). Importantly, prefrontal and parietal areas were active in both 220–280 ms and 540–600 ms time windows. These areas are thought to be of particular relevance to conscious experience (see [43] for review). Right inferior temporal cortex (ITG) and left motor cortex (PreCG) displayed significant activation only in the 220–280 ms time window. As all subjects responded using only their right hand, the left motor cortex was directly responsible for initiating button presses. The rivaling stimuli in this experiment were images consisting of complex patterns (see Figure 1), significant in this context because the right inferior temporal cortex is known to be particularly relevant for the processing of this type of stimulus [44], including during rivalry of complex patterns [45]. Activation of right motor cortex was observed during both 220–280 ms and 540–600 ms time windows.

**Table 1:** Statistical significance level for gamma-band sources in identified periods of scalp gamma activity and locations of peak activation used to seed the dipole source montage.

Source	Brodmann Area	MNI Coordinates (mm)			540–600 ms	220–280 ms
		*x*	*y*	*z*		
R PreC	7	22	−68	52	p<0.05	p<0.01
L DLPFC	8	−24	30	51	p<0.05*	p<0.01
R DLPFC	8	34	34	41	p<0.05*	p<0.05
L SFG	10	−17	66	8	p<0.01	p<0.01*
R SFG	10	19	66	8	p<0.01	p<0.01
L PreCG	6	−58	−5	37	n.s.	p<0.01
R PreCG	6	49	−5	36	p<0.01	p<0.01
R ITG	20	61	−27	−23	n.s.	p<0.05

**Abbreviations:** ***R***, right; ***L***, left; ***PreC***, precuneus; ***DLPFC***, dorsolateral prefrontal cortex; ***SFG***, superior frontal gyrus; ***PreCG***, precentral gyrus; ***ITG***, inferior temporal gyrus; ***n.s.***, not significant.

* indicates instances where the 3D rendered activation was ambiguous and confirmatory statistics were performed on peak voxels.

### Inter-regional gamma-band phase synchronization

**Figure 5:**
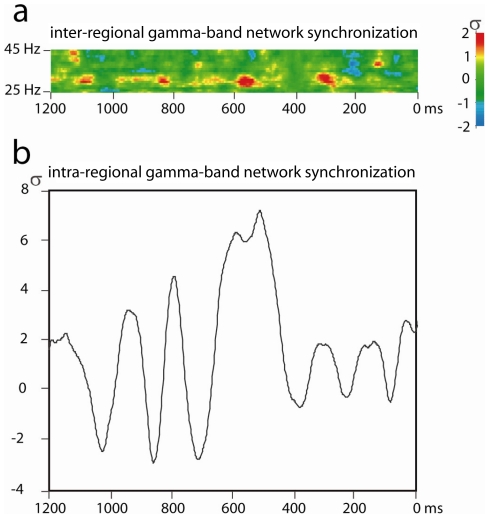
a) Time-course of averaged gamma-band phase synchronization (standardized PLV) between cortical regions identified by beamformer source localization preceding the onset of stable percepts. b) Time-course of averaged intra-regional neural synchronization preceding the onset of stable percepts (standardized amplitude from the analytic signal analysis) at centre frequency identified for inter-regional synchronization (33 Hz).

We hypothesized that gamma-band phase synchronization would be observed between cortical areas displaying increased gamma-band activation during the 220–280 ms and 540–600 ms pre-response windows. In our view, gamma synchronizations recurring at a theta rate represent the integration of relevant neural populations into large-scale ensembles. This entails functional integration across regions by means of gamma-band phase synchronization. Again, this synchronization would be expected relative to phase scattering of gamma rhythms in the period intervening between the 220–280 ms and 540–600 ms pre-response windows. To assess gamma-band phase synchronization between neural sources we extracted epoched time-series data from all sources identified by the beamformer analysis in the −220 to −280 ms and −540 to −600 ms time windows using a source montage (BESA 5.2; Megis Software). Phase locking values (PLVs) were then computed to assess inter-regional synchronization relative to the 370–430 ms baseline period. This analysis revealed gamma-band phase synchronization between many pairs of activated cortical regions in both the 220–280 ms and 540–600 ms time windows, and particularly in the 220–280 ms window related to the onset of a new percept (Figure 4). Moreover, the observed inter-regional gamma-band synchronizations appear to partake of an ongoing pattern of intra-regional synchronizations that recur at a frequency in the theta band. This is evidenced by the ordered procession of increases in inter-regional gamma-band synchronization in advance of button presses, which is accompanied by largely coincident increases in intra-regional gamma-band neuronal synchronization (Figure 5). The ongoing rhythm of both intra-regional and inter-regional gamma-band synchronization is consistent with a theta rate of roughly 4–6 Hz (see Figure 5).

### Modulation of gamma-band network activity by theta phase

**Figure 6:**
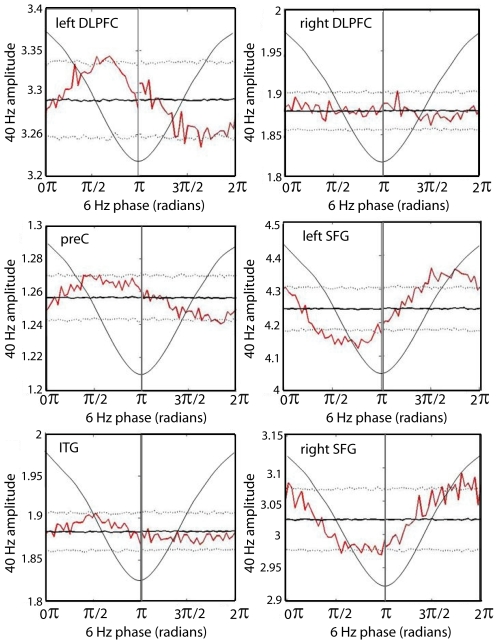
Theta-modulation of gamma amplitude. Dotted lines represent the 97.5 (top) and 2.5^th^ (bottom) percentiles and the dark black line indicates the mean of the surrogate distribution for each of the 60 bins of the theta cycle. Jagged red line denotes the mean non-normalized gamma amplitude in each bin. When the gamma amplitude was greater than or less than the surrogate line for two or more successive bins we considered the departure to be significant (*p*<0.05, two tailed). Left PreCG and right PreCG plots are not shown; they resemble the plot for right DLPFC and show no significant relationship between theta phase and gamma amplitude. Radians on the x-axis are in reference to a cosine wave, which is maximal at 0 radians; one cycle of a 6 Hz cosine wave (thin black line) is superimposed on the graph.

The above results suggest that the activation of a gamma-oscillatory network of cortical areas is modulated by the phase of theta-band oscillations within those brain regions. To test this hypothesis directly we examined whether gamma (40 Hz, actually 38–42 Hz filtered) z) Hamplitude was modulated by theta (6 Hz, actually 5.7–7.3 Hz filtered) phase in each of the cortical regions identified by beamformer analysis. In this analysis we took the theta phases and gamma amplitudes directly from the analytic signal over entire 1000 ms pre-response epochs and did not normalize them relative to the 370–430 ms baseline (see Methods). That baseline period, rather, was included in each epoch. This means that 6 theta cycles occurred in each epoch, although probably not exactly the same 6 cycles. Statistically significant (*p*<0.05, two-tailed) modulation of gamma amplitude by theta phase was found in bilateral SFG, left DLPFC, and right PreC (Figure 6). Modulation of gamma amplitude by theta phase was also apparent in right DLPFC and right ITG, but failed to reach statistical significance for at least two successive bins (see methods). We thus identified significant modulation of gamma activity by theta phase in four of the five areas involved in the recurrent gamma-oscillatory network time-locked to perceptual switching. Interestingly, the relationship of gamma amplitude to theta phase was virtually identical for the two SFG sites, and also for left DLPFC, right PreC and right ITG, but differed between these two groups by approximately π radians (180°). Moreover, neither of the peaks of gamma amplitude occurred at the theta trough, as found by Canolty et al [31] for 80–150 Hz amplitude, but rather peaks in both sub-groups occurred nearer to the zero-crossing of the theta oscillation about π/4 radians from the theta minimum (which would occur around±π).

**Figure 7:**
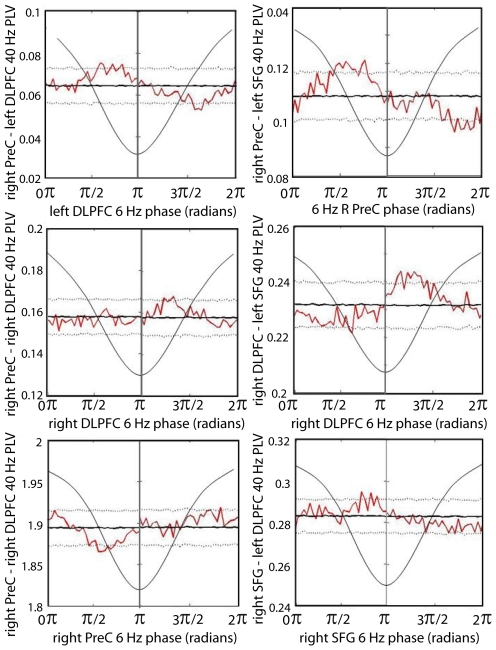
Theta-modulation of gamma-band inter-regional phase locking. Dotted lines represent the 97.5 (top) and 2.5^th^ (bottom) percentiles and the dark black line indicates the mean of the surrogate distribution for each of the 60 bins of the theta cycle. Jagged red line denotes the non-normalized gamma-band phase locking value in each bin. When phase-locking was greater than or less than the surrogate line for two or more successive bins we considered the departure to be significant (*p*<0.05, two-tailed). Radians on the x-axis are in reference to a cosine wave, which is maximal at 0 radians; one cycle of a 6 Hz cosine wave (thin black line) is superimposed on the graph.

Phase synchronization of gamma-band oscillations between cortical regions was also found to be modulated by theta phase. Table 2 and Figure 7 display the results of this analysis for all pairs that were active in both the 220–280 ms and 540–600 ms time windows. Again, in this analysis we used the same 1000 ms pre-response epochs and theta phases and gamma phase locking values were *not* normalized relative to the 370–430 ms baseline; rather their values in that baseline period were included in the analysis. As can be seen from Table 2, gamma-band phase synchronization was modulated by the phase of theta oscillations in at least one of the two regions involved for many of the region pairs. Importantly, synchronization of parietal and frontal areas was modulated either by parietal or frontal theta phase, or both, for all but the right superior frontal gyrus. Moreover, there was also significant modulation of phase locking among frontal areas by theta phase in one or both areas of a pair. Modulation of gamma-band phase locking by theta phase was also found between R PreCG and PreC and between R PreCG and frontal sources. These modulations involving R PreCG were, however, generally less pronounced that synchronization between prefrontal and parietal regions. As was found in the case of the amplitude modulations, these phase synchronization modulations did not always coincide with either a peak or a trough in the theta rhythm. They are evidence, however, of a strong association between inter-regional gamma-band synchronization and the phases of ongoing theta-band cortical rhythms.

**Table 2:** Summary of gamma-PLV modulation by theta phase and inter-regional interaction between theta phases.

Source Pair	PLV by 1^st^?*	PLV by 2^nd^?	Theta-theta?	Theta phase relationship
R SFG-R DLPFC	Yes	Yes	Yes	3π/4
R DLPFC-L DLPFC	No	No	Yes	3π/4 & π/4
R DLPFC-L SFG	Yes	Yes	Yes	3π/4
L SFG-L DLPFC	No	No	Yes	π/4 & 3π/4
L DLPFC-R SFG	Yes	No	Yes	π/4
L SFG-R SFG	No	No	Yes	3π/4
R PreC-R DLPFC	Yes	Yes	Yes	π/4
R PreC-L DLPFC	No	Yes	Yes	3π/4 & π/4
R PreC-R SFG	No	No	Yes	π/4
R PreC-L SFG	Yes	Yes	Yes	π/4
R SFG-R PreCG	No	Yes	Yes	3π/4
R DLPFC-R PreCG	No	No?	Yes	3π/4
L SFG-R PreCG	Yes	Yes	Yes	3π/4
L DLPFC-R PreCG	No	Yes	Yes	3π/4
R PreC-R PreCG	No	Yes	Yes	π/4

**Abbreviations:** ***R***, right; ***L***, left; ***PreC***, precuneus; ***DLPFC***, dorsolateral prefrontal cortex; ***SFG***, superior frontal gyrus; ***PreCG***, precentral gyrus.

* ***Yes*** means significant at least at two successive theta phases; ***No*** means never significant.

PLV by 1^st^(2^nd^) indicates that PLV was modulated by the 1^st^(2^nd^) of the regions listed in the source pair. Theta phase relationship indicates the phase relationship between theta oscillations in the two analyzed regions.

**Figure 8:**
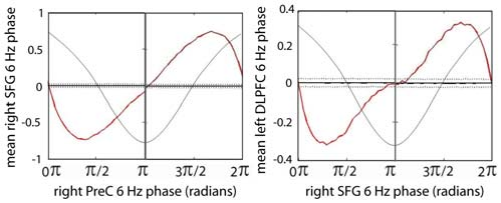
Two examples of theta-theta phase relationship. Dotted lines represent the 97.5 (top) and 2.5^th^ (bottom) percentiles and the dark black line indicates the mean of the surrogate distribution for each of the 60 bins of the theta cycle. Jagged red line denotes the mean theta phase of the source indicated on the y-axis in each bin of the theta source indicated on the x-axis. When the mean phase was greater than or less than the surrogate line for two or more successive bins we considered the departure to be significant (*p*<0.05, two-tailed). Radians on the x-axis are in reference to a cosine wave, which is maximal at 0 radians; one cycle of a 6 Hz cosine wave (thin black line) is superimposed on the graph.

The beamformer and phase locking analyses uncovered a gamma oscillatory network which is recurrently activated and integrated at a theta rate. Activation and inter-regional synchronization in this network is modulated by theta phase but, paradoxically, the precise relationship to theta oscillations differs between sources and source pairs. This suggests that theta rhythms differ in phase across the activated regions. To investigate this we analyzed theta phase relationships between activated cortical areas. Figure 8 and Table 2 display the results of analysis of these data for the relationship between non-normalized theta phases in the various region pairs. Clearly there is a strong relationship for all of them, akin to significant phase locking, but the various rhythms clearly do not correspond to a single, trans-cortical rhythm. Rather, there is a tendency for the theta rhythms in the various brain areas to be phase locked with varying amounts of phase difference. This makes it possible for gamma rhythms locked locally to theta phase in their own region to also be locked across regions, and to follow the theta rhythms in inducing perceptual changes.

## Discussion

### Large-scale oscillatory networks and perceptual consciousness

We have demonstrated that button presses indicating the onset of a new percept in binocular rivalry of complex patterns are preceded by time-locked bursts of gamma-band activation that recur at a theta rate. Source imaging of gamma-band activations 220–280 ms and 540–600 ms prior to responses revealed a recurrent network of prefrontal and parietal areas consistent with numerous fMRI investigations implicating these regions in perceptual transitions in binocular rivalry and in alternations of bistable figures [see 43], [46 for reviews]. Critically, this prefrontal-parietal network has been shown to be engaged when changes in a visual scene are detected, confirming that it is relevant to the updating of perceptual consciousness in general and not only to bistable perception [47]. The similarity of our source solution to cortical networks identified by hemodynamic neuroimaging also indicates that observed gamma-band activations are unlikely to arise from ocular artifacts such as microsaccades, which would be localized to the eyes and rejected from our solution. We also demonstrated that the cortical sources of gamma activity in the 220–280 ms and 540–600 ms pre-response time windows display increases in inter-regional gamma-band phase synchronization, thereby integrating those regions into a transient functional network. This result supports the view that consciousness emerges as a product of large-scale brain integration implemented by synchronization of relevant neural populations in the gamma-band [6], [7]. We interpret this as reflecting the selective integration of information represented in relevant cortical regions into a large-scale assembly that constitutes a global workspace for consciousness [1], [3]. Periodic activation of, and integration within, this network would thus correspond to the formation of a new large-scale assembly defining conscious contents and, in the context of the present inquiry, a window of time during which the onset of a new percept occurs.

In this view, prefrontal cortex is an essential component of the ‘consciousness network’ because of its relevance for integration generally and for self-awareness, whereas parietal cortex is critical as it contains a multimodal representation of space in which the representation of self is located relative to the perceptual world [43], [48], [49]. The precuneus, identified in the present study, is also specifically associated with the experience of agency, mental imagery, episodic memory retrieval, and first person perspective taking, and has abundant connections with prefrontal cortex, further implicating it in perceptual experience [see 48 for review]. The spread of surface-rendered activation on the cortical surface suggests that parietal activity may have also extended to other regions relevant for perceptual space. The integrated intersection of ‘observing’ and ‘representing’ faculties situated in prefrontal and parietal cortex, respectively, may thus reflect the substrate of perceptual consciousness [4], [50].

Interestingly, primary visual cortex was not identified as a generator of gamma rhythms time-locked to perceptual switching. Although required for perceptual experience, neuroanatomical and psychophysical data suggest that we do not directly experience activity in striate cortex [43], [51]. This supports the view that the large-scale oscillatory network detailed here is essentially related to perceptual experience itself, and not to those unconscious functions that give rise to changes within it. It is known that perceptual transitions in binocular rivalry involve primary visual cortex [see 52 for review], and are associated with changes in gamma-band synchronization within primary visual cortex [53], [54]. It is a matter of some debate, however, whether activity in primary visual cortex is relevant to conscious experience *per se*, or whether lesions of this region simply disturb consciousness by disrupting the flow of information to higher brain regions [55].

We found that the recurrent gamma-oscillatory network identified in this study was modulated at a theta frequency, consistent with previous studies of endogenous oscillatory synchronization time-locked to perceptual switching in binocular rivalry [25]. This supports the hypothesis that theta-modulated gamma-band synchronizations are essentially related to perceptual experience and define discrete ‘frames’ of consciousness, consistent with results from attentional blink experiments and those investigating coherent perception of visual images [19], [24]. The distribution of dominance durations in our study, consistent with findings from previous studies, suggests that perceptual switching did not occur on every theta cycle. This indicates that the theta cycle determines when a new perceptual experience *can* occur, but that the content of each ‘frame’ of consciousness does not need to differ from that of its predecessor (see Figure 1). For example, continuous viewing of a single unchanging stimulus will yield a procession of theta cycles in which the content represented on each cycle remains the same. Since perceptual transitions are not manifest on each theta cycle, it is apparent that some additional mechanism is at play, and determination of what induces perceptual transitions on particular cycles represents an important question for future study. It seems very likely, however, that a complete theta cycle is necessary, if not sufficient, for the onset of a new perceptual experience. This is evidenced by the finding that when stimuli are presented at speeds above the theta rate not all of these stimuli would result in perceptual experience as there would not be enough ‘frames’ to represent each one (attentional blink). Although discreet moments of perception can only occur at a certain rate, as demonstrated by the attentional blink phenomenon, subjective consciousness is seamless and continuous rather than presenting itself as a sequence of discrete conscious moments. The results presented here suggest a similar arrangement, as perceptual consciousness is updated by a periodic mechanism but is experienced as a continuous and stream of consciousness. The precise physiological mechanisms responsible for coherent transitions from one conscious frame to the next and the integration of discrete intervals into a coherent stream of conscious experiences remain unclear. Consciousness vectors, however, analogous in function to visual motion vectors in area V5/MT and arising from prefrontal function, have been proposed to underlie this function [56], [57].

Illustrative of the relationship between these oscillatory mechanisms and dominance durations is the fact that gamma-band activation of right ITG and left PreCG occurred in the 220–280 ms pre-response window yet was absent in the earlier 540–600 ms pre-response interval. This suggests that it was during this period of gamma-band synchronization that a new percept emerged since the incorporation of the ITG, known to be involved in the processing of higher-order visual patterns, could have integrated elements of the emerging percept into the large-scale gamma-oscillatory neural assembly defining conscious contents [44]. Supporting this notion are studies that used implanted electrodes and fMRI to demonstrate that stimulus-specific activations of inferior temporal cortex are associated with awareness of visual stimuli similar to those used in the present paradigm [44], [45]. Moreover, activation of left PreCG only during the 220–280 ms pre-response period suggests that the initiation of the subsequent behavioural responses occurred during that interval. In this view, the essential prefrontal-parietal gamma-oscillatory network uniquely subsumed areas responsible for processing complex visual forms and patterns (right ITG) and areas required to initiate a response (left PreCG) during the −220 to −280 ms period, indicating that during this interval a new image was incorporated into visual experience and a button press was initiated. Significant activation of right ITG and left PreCG was not observed in the 540–600 ms interval, presumably because this cycle more likely does not correspond to the onset of a new percept requiring a response, as evidenced by the average duration of dominance periods (see Figure 3). Further buttressing this interpretation is the finding that whereas cortical regions within the recurrent prefrontal-parietal consciousness network tended to show strong, statistically significant modulation of gamma amplitude by theta phase, ITG and PreCG did not. This suggests that these regions are incorporated into the gamma oscillatory network only on the minority of theta cycles on which a perceptual switch occurs and a response is required. Interestingly, right PreCG was active in both 220–280 and 540–600 pre-response time windows and showed evidence of theta modulation of gamma-band phase locking with parietal and prefrontal cortex. This modulation locking was less pronounced than was generally observed between prefrontal-parietal and prefrontal-prefrontal source pairs, however, and no significant theta modulation of gamma amplitude was observed in right PreCG. As such, future research will be required to determine what role, if any, the right PreCG plays in perceptual transitions.

We found that both the amplitude and the inter-regional synchronization of gamma oscillations in the identified network of cortical regions were coupled to the phases of theta oscillations in those same areas. This confirms that the cortical theta rhythms modulate the periodic assembly and disintegration of gamma-oscillatory neural coalitions time-locked to the onset of new percepts in binocular rivalry. Interestingly, however, maximal gamma amplitude in bilateral SFG was occurred roughly ½ theta cycle (about 83 ms) out of phase with left DLPFC, PreC and ITG (see Figure 6). This result was unexpected, given that high gamma-band oscillations have been previously found to be maximal during the trough of ongoing theta oscillations [31]. Our analysis, centred within a lower 35–45 Hz frequency range, suggests that coincident gamma activations in different cortical regions can be locked to different theta phases. Moreover, a similar result was found for inter-regional synchronization in the gamma band: the synchronizations were modulated differently by theta phase in the different region pairs, sometimes by theta phase in only one of the regions, but also sometimes by different theta phases in the two regions. An example of the latter is the right PreC and right DLPFC pair, where phase locking value was maximal at a right DLPFC theta phase of around 5π/4, whereas it was maximal at a right PreC theta phase of about 7π/4. Importantly, the theta phases in these two regions tended to differ by about π/4, indicating that the gamma synchronization maxima were locked to different theta phases each both regions. Moreover, theta phases in all source regions tended to be locked with each other, either with a phase difference of about π/4 or about 3π/4 (see Table 2). This result confirms that periodic gamma synchronization is recurring at a theta rate within regions comprising the prefrontal-parietal ‘consciousness network,’ and that the local gamma activations and inter-regional synchronizations are modulated by theta phase, but indicates that theta oscillations differ in phase across cortical regions. Interestingly, left DLPFC displayed a propensity for theta phase differences with three other regions of both π/4 and 3π/4, suggesting that left DLPFC must be alternating between two relatively stable and roughly equally strong phase relationships with other areas. Beamformer source localization, together with assessment of phase locking and instantaneous amplitude in source-reconstructed time-series, also indicates that these regions show recurrent coincident gamma activation and inter-regional synchronization (see Figures 4 and 5). Thus our hypotheses are confirmed that recurrent activation and integration of a gamma-oscillatory network of cortical regions is time-locked to the onset of new visual percepts in binocular rivalry, and that the formation and dissolution of these neuronal complexes are phase-locked to ongoing cortical theta rhythms. Our results suggest, however, that an overarching cortical theta ‘cycle’ would in fact consist of multiple synchronized oscillators which each maximized phase-locked gamma activity during the same time intervals, despite phase offsets. In order for such theta oscillations to maintain periodic gamma activation across cortical regions, it must be the case either that the theta oscillators are interacting across cortical regions, or that their synchrony is maintained by some mechanism exterior to the oscillators themselves. The finding that coincident gamma activation within different cortical regions is locked to different theta phases suggests that the interplay between these slow and fast rhythms is, at least in some circumstances, more complex than was previously imagined. Further research will be required to better illuminate the nature and function of these cross-frequency relationships across various experimental conditions, as well as across frequency ranges within the gamma band [see 31]. Specifically, the generality of these mechanisms will need to be established. It is not clear how the network mechanisms revealed by perceptual transitions during binocular rivalry correspond to cortical dynamics across the wider context of mental life. The temporal profile and characteristic patterns of oscillatory synchronization during the attention blink suggest that theta-gamma mechanisms implement independent frames of perceptual experience, but it is not clear how such activity would be affected by more complex and realistic perceptual conditions or externally induced alternations in binocular rivalry. For example, it has been shown that salient stimuli can reset the phase of theta oscillations [58], suggesting that the timing of gamma oscillatory mechanisms underlying perceptual experience may be affected by environmental demands.

Interposed between recurrent gamma-band synchronizations are periods during which gamma rhythms become relatively desynchronized. These intervals likely play an important role in ongoing cortical network dynamics underlying the stream of perceptual consciousness. Transient desynchronization of gamma oscillations has been shown to occur between periods of transient task-relevant synchronization [19]. This process, known as ‘phase scattering’ is understood as a mechanism by which existing task- and/or percept-dependent neural coalitions are terminated. This periodic dissolution of functional connectivity allows new assemblies of synchronously oscillating neural populations to emerge in order to code the features of a new percept or integrate processing elements required for new tasks. In the present study these periods, such as the 370–430 ms pre-response interval used as the baseline in some of the analyses, likely play an important role in implementing the stream of perceptual consciousness by ending each ‘frame’ of perceptual experience. Notably, the beamformer source localization techniques we employed here operate by localizing the sources of activity within one time window relative to activity in a second time window of equivalent length. Accordingly, localization of 35–45 Hz activity from −220 to −280 ms relative to −370 to −430 ms would provide the same source solution as the opposite analysis, except with opposite valence of activation/deactivation. Viewed through this relativistic lens, our results demonstrate a network of prefrontal and parietal cortical regions that cycles between gamma synchronization and desynchronization, phase locked to cortical theta rhythms. We propose that these oscillatory network dynamics provide a temporal structure governing the emergence and abolition of discrete moments of experience.

Electroencephalographic investigation of working memory, a faculty integrally associated with perceptual consciousness, has yielded three additional key findings suggestive of a fronto-parietal network associated with theta-gamma oscillatory mechanisms that may implement a ‘global workspace’ for the retention and manipulation of to-be-remembered material. During the retention interval (1) increased gamma-band activation is observed at frontal and parietal electrodes, (2) coupling of theta-band and gamma-band oscillations is observed at frontal and parietal electrodes, and (3) theta-band and gamma-band synchronization is enhanced between frontal and parietal electrodes [34], [59], [60]. Results such as these, together with behavioural indices of working memory suggestive of theta-modulated gamma-band mechanisms (gamma/theta = individual working memory span; 40/6≈7), have led to the hypothesis that items are maintained in working memory by assemblies of representational cell coalitions that are refreshed at a gamma frequency during each theta cycle [see 61]. Such results are consistent with accumulating evidence that cross-frequency synchronization may be a key mechanism for the organization of dynamic activity in the nervous system [62].

Interestingly, neuroimaging results are now converging on an anatomical network relevant to general intelligence that is highly correlated with working memory span and is centered in frontal and parietal cortex [63]. On such evidence it could be speculated that the conscious workspace, of which working memory is an application, emerges from an essential fronto-parietal network that achieves functional conjunction via theta/gamma oscillatory mechanisms, and that this essential constellation of brain regions is expanded to include other cortical regions specifically relevant to perceptions and tasks permeating consciousness at that moment. This notion is supported by the finding that attention, understood as the gateway to consciousness, biases information for inclusion in a large-scale gamma-synchronous network [35]. This network workspace may allow for the flexible manipulation of information and flexible expression of learned behaviour, considered to be one of the cardinal cognitive attributes of conscious experience [64]. Accordingly, our data suggest that consciousness should not be conceived as what remains when no task-relevant region attributable to specific faculties is removed, but rather as the integrated amalgamation of all momentarily relevant faculties into a unitary constellation: the observer; the intentional actor.

The results detailed here are consistent with a more general understanding of how mental representations arise from the activity of distributed neural groups. Donald Hebb proposed that memory traces and mental representations were implemented by connectivity within a distributed network of neurons, and that the selective re-ignition of this assembly would be tantamount to recognition and/or recall [65]. This notion is supported by evidence that the perception of a familiar object, even in a degraded form, is also associated with both intra-regional and inter-regional gamma-band synchronization [18], [66]. The Hebbian outlook, coupled with a more modern understanding of functional cortical anatomy, would predict that cortical regions relevant to the representation of a memory would be activated and functionally integrated during recognition and recall. Indeed, it has been shown that recognition of a complex object is associated with gamma-band activation in frontal, parietal and temporal cortex, between which enhanced gamma-band phase synchronization and bidirectional causal interaction are observed [11], [67]. In light of the present results, this could be viewed as the integration of temporal areas responsible for the processing of complex images into the fronto-parietal gamma-oscillatory network integral to perceptual experience. It is notable that schizophrenia, historically considered to be a disorder of consciousness and characterized by cognitive fragmentation, is associated with abnormal theta and gamma band oscillatory activity during cognitive processing [see 68], [69 for reviews]. The essential relationship between theta-gamma mechanisms and consciousness is also highlighted by a possible common neuropharmacological substrate. The bursting of cholinergic projections to cortex from basal forebrain is found only in wakefulness and REM sleep, central nervous system states associated with consciousness, and theta and gamma activity are correlated with bursting in these neurons [70]. Such results demonstrate that gamma-band neural synchronization is a fundamental mechanism for mental representation, and that its disturbance results in alterations of conscious experience.

### Implications for free will

Our results may also have implications for the interpretation of what are perhaps some of the most disconcerting findings in human neuroscience. Benjamin Libet used an ingenious experimental paradigm to provide evidence that the conscious intention to initiate a movement was substantially (approximately 300 ms) preceded by the onset of the readiness potential, or RP, a buildup of scalp-recorded electrical activity originating in the motor cortices, taken to indicate the initiation of a behavioural response [71], [72]. Libet considered this to be evidence that unconscious impulses were the true cause of action, and that the conscious experience of intent to move occurred later and was antedated in our experiential model of the world. The interpretation of this result has been the subject of great debate and scrutiny. Most damaging has been the criticism that it is not possible, at least in any straightforward way, to compare the timing of conscious perceptions to the timing of their external causes, such as the perception of the stimulus that Libet used to measure the timing of action intention [73].

Although the experimental paradigm used in the present study was not designed to directly address the temporal relationship between movement and the will to act, our results provide a unique vista on the relation of gamma-band activity and synchronization to consciousness and behaviour. Our data indicate that gamma-band activation of left PreCG, presumably involved in the generation of a behavioural response to a change in consciousness, is uniquely present in the 220–280 ms pre-response period of activation, and is notably absent in the preceding 540–600 ms window. Moreover, left PreCG is integrated into the prefrontal-parietal network by means of inter-regional gamma-band phase synchronization during the same cycle that ITC is activated and integrated into this network. These observations suggest that initiation of behavioural responses signaling the onset of new conscious percepts, at least as indexed by gamma-band activity, precede action and occur at reaction times consistent with those observed in complex perceptual tasks. This is consistent with evidence that gamma-band oscillations in motor cortex are relevant to the initiation and control of movement [74]. This means, contrary to Libet's conclusions, that at least one neural indicator of the timing of conscious experience is compatible with the view that consciousness is relevant to the initiation of volitional action. Initiation of a behavioural response coincides with perception of a new image, indexed by activation and network integration of ITC, indicating that conscious experience is directly related either to the initiation of a behaviour or to the awareness of its initiation. By relating ongoing internal processes to behaviour, rather than attempting to correlate the internal perception of an external event with the timing of behaviour, we have overcome a critical confound of Libet's paradigm. A liberal interpretation of our results would suggest that (1) consciousness, or biological processes directly underpinning consciousness, may indeed control behaviour, and (2) the experience of volition is not antedated. Such interpretations, however, are speculative as our paradigm is not ideal for the evaluation of these hypotheses. It must also be acknowledged that neural activity in other frequency ranges might precede that in the gamma band, and might be more directly related to the initiation of a voluntary response. Thus, our findings simply suggest an intriguing avenue for future research into the chronometry of experience and its implications for free will.

## Methods

### Subjects and experimental paradigm

Data were recorded from 14 subjects with normal or corrected-to-normal vision. Subjects viewed rivaling stimuli through a mirror stereoscope while EEG was recorded from 59 electrodes at standard 10–10 locations plus 3 at nonstandard locations below the inion, using an isolated bioelectric amplifier (SA Instrumentation, Inc., San Diego, CA). Scalp recordings were referenced to the right mastoid electrode. Electrodes placed above and beside the right eye were recorded bipolarly in order to identify and remove ocular artifacts. Impedances were kept below 15 kΩ (sufficient as amplifier input impedances were >2 GΩ). Stimuli were complex images (a blue butterfly on a yellow background and orange maple leaves on a blue background, each measuring 5.3° wide by 5.6° tall viewed at a distance of approximately 73 cm from the eye's nodal point) with small red circular dots in the centres and white borders surrounding each image to maintain and judge alignment (see Figure 1). Contrast and luminance were chosen from a palette to yield approximately equal full dominance intervals for the two percepts and were relatively high (standard colours in Adobe Illustrator ©). These stimulus parameters yielded a modal full dominance interval around 550 ms.

Subjects were seated and a chin rest was used to maintain head position throughout the experiment. Subjects were instructed to depress buttons using two fingers on their right hand to indicate which of the rivaling images, if any, completely dominated perception at any given time. Specifically, subjects were instructed to depress button 1(2) to signal onset of dominance for left(right) eye stimulus, continue to hold down button 1(2) as long as the left(right) eye stimulus dominated, and to release button 1(2) to signal loss of perceptual dominance for left(right) eye stimulus. This produced a continuous record of the onset and offset of periods of perceptual dominance. Prior to recording, subjects received training to identify periods of complete dominance and to check the alignment on left and right eye stimuli. After training, subjects performed in 12 blocks lasting 4 minutes each. Between each block subjects were given the opportunity to rest, after which they were presented with a screen directing them to check the alignment of the stimuli before commencing the next block. Rest and the realignment periods were terminated by responses from subjects. EEG data were filtered at 0.1–100 Hz, amplified with a gain of 20,000, digitized at 500 Hz, and stored for off-line analysis. All subjects gave written informed consent before participating in the experiment. The protocol was approved by the UBC Behavioural Research Ethics Board.

After the data were recorded, behavioural indices of perceptual switching were computed to determine the number of epochs of stable perceptual dominance lasting 700 ms or longer. Epoch length was defined as the time between a button press signaling the onset of a period of complete perceptual dominance by one of the two images and the release of that button signaling the termination of complete perceptual dominance by that image. An interval of complete perceptual dominance was defined as a period wherein the perception of one stimulus was uninterrupted, either by perception of the other stimulus or by an ambiguous percept containing elements from both images. Subjects who did not display stable patterns of rivalry during their first session were excluded from the study. Subjects who did display stable patterns of rivalry but lacked a sufficient number of stable percepts for analysis were asked to return for a second, and if necessary, third recording session. Ten of the 14 subjects displayed a pattern of stable periods of complete perceptual dominance and their data were retained for further analysis.

### Beamformer source localization

Epochs time-locked to button presses signaling the onset of a new stable percept were extracted from 1300 ms prior to the press until 300 ms after it. Stable percepts were defined as those lasting 700 ms or longer. Epochs containing ocular and nonocular artifacts were rejected using the automated artifact rejection algorithm implemented in the Brain Electrical Source Analysis software suite (BESA 5.2; Megis Software). One subject's data were excluded from analysis because of excessive contamination by artifacts. Data from the remaining 9 subjects were used for the subsequent electrophysiological analysis (mean age 23.3; 3 female). Data from these subjects yielded 3281 epochs of stable perceptual dominance with approximately equal incidence of left eye and right eye dominance (1805 left eye; 1476 right eye).

Scalp data for individual participants were transformed into the time-frequency domain from 10 to 60 Hz using the complex demodulation procedure implemented in BESA with a time-frequency sampling of 5 Hz/10 ms [see 75]. Time windows of interest were selected by averaging the time-frequency data across participants and across a subset of electrodes that encompassed the fronto-parietal regions where theta-modulated gamma-band activity was expressed most clearly (F1, F3, F5, F2, F4, F6, FC1, FC3, FC5, FC2, FC4, FC6, C1, C3, C5, C2, C4, C6, CP1, CP3, CP5, CP2, CP4, CP6, P1, P3, P5, P2, P4, P6). The lowest gamma power occurred approximately 400 ms pre-response, with peaks of gamma activity in the 35–45 Hz range occurring on either side of this power decrease at approximately 220–280 ms and 540–600 ms pre-response (see Figure 3). The topographical distribution on the scalp of the 35–45 Hz activity in each of the two time intervals of interest were plotted, relative to the amplitude of the activity in the same frequency band during the baseline period, in Figure 3a. Increases in intra-regional gamma-band synchronization were widely distributed over the scalp, largely over frontal, central and parietal scalp sites.

To identify cortical loci expressing intra-regional gamma-band synchronization time-locked to button presses indicating perceptual switching, the Multiple Source Beamformer implemented in BESA was applied to data from each subject using a standard realistic head model. This method has been demonstrated to be effective for localization of sources of oscillatory activity within highly specific time-frequency windows [76]. Beamformer analysis estimates the contribution of a given voxel in the brain to activity recorded on the scalp within a specified time-frequency window by minimizing contributions from all other voxels in source space, thereby implementing a spatial filter to identify neural generators of scalp activation [77], [78]. This analysis requires a baseline period of the same duration as the time window of interest. Therefore we applied the beamformer to 35–45 Hz activity occurring 220–280 ms and 540–600 ms pre-response, relative to an intervening 370–430 ms baseline in the same frequency range. Beamformer source reconstructions were obtained separately for each participant in each of the time intervals of interest. Non-parametric statistical analysis was then performed using functional neuroimaging analysis software (AFNI) [79]. Statistically significant (*p*<.05 or greater) sources of activity were then displayed on a three-dimensional rendered standard brain. The statistical results indicated the presence of bilateral generators in both DLPFC and SFG. Due to the proximity of the prefrontal sources, however, it was unclear whether there were actually four distinct source locations or if any of the statistically significant sources reflected the spread of activity from nearby neighboring sources. To ensure that we were accurately identifying source locations for the subsequent PLV analyses, we examined the average beamformer output for all participants, without statistical analysis, confirming that the DLPFC and SFG sources were distinct from one another. Ambiguity of statistical significance for reasons of source proximity or rendering orientation was apparent in the group surface-rendered data in the cases of the right DLPFC and left DLPFC in the 540–600 ms window and left SFG in the 220–280 ms window (see Figure 4). In these cases, it was unclear whether these foci of gamma identified activity in source space were producing statistically significant activation on the surface-rendered image, or if the spread of significant activation attributable to only one source. To confirm that each of these sources was significant, the voxel representing peak activation for each source in question was subjected to statistical analysis (see Table 1). While useful in resolving such scenarios of ambiguity, the approach of testing peak voxels was not employed as a primary assessment because (1) it does not provide an accurate representation of the anatomical spread of the generator, thereby obfuscating the functional significance of the cortical source, and (2) it would require the added assumption that activity of the peak voxel was essentially similar to that of adjacent cortex.

### Inter-regional phase locking

In order to assess synchronization within and between cortical sources of gamma-band activation time-locked to perceptual switching in binocular rivalry, we extracted epoched broadband time series using a source montage in which locations were assigned to coordinates of all activational peaks for statistically significant sources (Table 1; BESA 5.2; Megis Software). Notably, the algorithm we employed creates a spatial filter for each source that excludes contributions from any other modeled sources, as well as limiting the contributions of noise and non–modeled activity to the source activity [see 80 for algorithm]. The sources thus are equivalent to virtual electrodes in the brain with approximately 3–4 cm diameter [80]. Epoched broadband signals computed from the modeled sources were band-pass filtered digitally at 1 Hz intervals Hz (passband = *f*±0.05*f*, where *f* represents the filter frequency). We then calculated the analytic signal 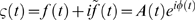



of the filtered waveform for each epoch, *f*(*t*), where 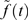 is the Hilbert transform of *f*(*t*) and 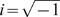, to obtain the instantaneous phase, *φ(t)*, and amplitude, *A*(*t*), at each sample point. Envelope amplitudes obtained in this manner and standardized as described in the next paragraph were used as indices of intra-regional neural synchronization.

We measured inter-regional phase synchronization by calculating phase-locking values. PLVs were computed from the differences of the instantaneous phases for pairs of time series from the reconstructed gamma-band sources, for example, sources *j* and *k*, at each point in time, *t*, across the *N* available epochs [81]: 
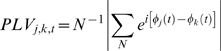



PLVs take on values between 0 (random phase difference, no phase locking) and 1 (constant phase difference, maximum phase locking). Our goal was to characterize inter-regional gamma-band synchronization occurring during periods of gamma activation time-locked to button presses indicating perceptual switching, relative to intervening phases of relative gamma desynchronization. This approach was employed to directly test the hypothesis that windows of gamma activity correspond to structured, synchronously oscillating neural ensembles corresponding to the construction of integrated experiences, whereas interposed desynchronizations reflect network dissolution. Accordingly, PLVs were calculated for each time point in the 220–280 ms and 540–600 ms windows. These values were standardized relative to the intervening 370–430 ms baseline. Standardization was accomplished by subtracting the mean baseline PLV from the PLV for each data point and dividing by the standard deviation of the baseline PLV. The resulting PLV_z_ scores indicate standardized changes from the average baseline PLV.

To assess the statistical reliability of changes from baseline in inter-regional synchronization and desynchronization we employed the surrogate statistical method [81]. To this end we scrambled the epochs and computed PLVs for the scrambled data for each frequency and time point combination for each pair of sources. The resulting PLVs were then standardized relative to the (scrambled) 370–430 ms pre-response baseline. This process was repeated 200 times to create a surrogate distribution for the relevant standardized PLV. The percentile rankings of real data from the normalized PLV values within the surrogate distributions were used to assess the statistical significance of observed changes in synchronization. A change in gamma-band synchronization was considered to be significant between a pair of cortical regions activated in either the 220–280 ms or 540–600 ms time windows at *p*<0.01 (two-tailed) if the real PLV was higher than all 200 data surrogates, and significant at *p*<0.05 (two-tailed) if the real PLV was greater than 195 of the data surrogates. To ensure confidence and conservatism in the reporting of these results, synchronizations were required to persist for a minimum of 25 ms (one cycle at 40 Hz and 12 data points at the 500 Hz sampling rate) and encompass multiple frequencies within the specified time-frequency window to be considered significant. Furthermore, in rare cases where significant desynchronization was found within the same time-frequency window as synchronization, the synchronization was not reported. The frequency window for assessment of inter-regional synchronization encompassed the 30–45 Hz frequency range, as the overall pattern of gamma-band synchronization was concentrated in that window (see Figure 5), although many individual source pairs were synchronized in the 35–45 Hz range used in the beamformer analysis.

### Theta modulation of gamma amplitude and inter-regional phase locking

To test the hypothesis that gamma-band activations were modulated by the phase of ongoing theta oscillations, cross-frequency coupling between theta phase (6 Hz) and gamma amplitude (40 Hz) was analyzed for each of the eight sources identified using beamformer analysis. Theta phase and gamma amplitude were obtained using digital filtering and the Hilbert transform, as described above, for a period beginning 1000 ms prior to button presses indicating the onset of a new period of perceptual dominance and ending with the button press. This provided time series data for theta phase and gamma amplitude for each analyzed epoch. Values in the cross-frequency analysis were not standardized relative to the baseline period. Gamma instantaneous amplitude was sorted according to theta phase, which was divided into 60 bins of 0.105 radians width, and mean gamma amplitude within each phase bin was calculated. The statistical significance of modulation of gamma amplitude by theta phase was tested by calculating a surrogate distribution obtained by creating 1000 sets of randomly-shuffled theta-phase time series and computing the mean gamma amplitudes within each phase bin in the same way. Modulation of gamma amplitude by theta phase was considered statistically significant (*p*<0.05, two-tailed) if the mean gamma amplitude was above the 97.5^th^ percentile, or below the 2.5^th^ percentile of amplitudes in the surrogate distribution for at least two adjacent bins.

Assessment of the modulation of inter-regional gamma-band phase locking was accomplished in a similar manner. The Hilbert transform was used to obtain the phases of non-normalized 40 Hz and 6 Hz signals from pairs of sources during 1000 ms epochs preceding button presses indicating the onset of a new perceptual dominance period. For each analyzed source pair, epochs were sorted according to the theta phase of one of the sources. This produced 60 bins, each 0.105 radians in width, and each containing 27,342 pairs of gamma phases for each source ((3281 epochs×500 time points)/60 bins). PLVs were then calculated in the manner described above within each bin, producing an index of the amount of gamma-band phase synchronization between a pair of sources typical during a particular segment of ongoing theta oscillations in one of the analyzed sources. Statistical significance was assessed by creating a surrogate distribution of 1000 sets of shuffled theta-phase time series and calculating gamma-band PLV in each phase bin in the same way. We considered modulation of inter-regional gamma-band phase locking by theta phase to be statistically significant (*p*<0.05, two-tailed) if the PLV was above the 97.5^th^ percentile, or below the 2.5^th^ percentile of the surrogate distribution for at least two adjacent bins.

The relationship between theta phases in pairs of brain regions was analyzed for the same 1000 ms epochs similarly to the analysis of the theta phase-gamma amplitude coupling. For theta phase, the epochs were sorted by the theta phase of one of the two regions in a pair, 60 bins 0.105 radians in width were created, and the mean theta phase in the other region was calculated for each bin. Statistical significance was assessed by creating a surrogate distribution of 1000 sets of shuffled theta-phase time series for a given region and calculating mean theta phase for the other region in each phase bin in the same way. We considered modulation of theta phase in one region by theta phase in another region to be statistically significant (*p*<0.05, two-tailed) if the mean theta phase was above the 97.5^th^ percentile, or below the 2.5^th^ percentile of the surrogate distribution for at least two adjacent bins.

## References

[1] Dehaene S, Naccache J (2001). Towards a cognitive neuroscience of consciousness: basic evidence and a workspace framework.. Cognition.

[2] Baars BJ (1997). In the theatre of consciousness.. J Consc Studies.

[3] Tononi G (2004). An information integration theory of consciousness.. B M C Neurosci.

[4] Crick F, Koch C (2003). A framework for consciousness.. Nat Neurosci.

[5] Başar E (2006). The theory of the whole-brain-work.. Int J Psychophysiol.

[6] Engel AK, Singer W (2001). Temporal binding and the neural correlates of sensory awareness.. Trends Cogn Sci.

[7] Varela F, Lachaux JP, Rodriguez E, Martinerie J (2001). The brainweb: Phase synchronization and large scale integration.. Nat Rev Neurosci.

[8] John ER (2002). The neurophysics of consciousness.. Brain Res Rev.

[9] Fries P (2005). A mechanism for cognitive dynamics: Neuronal communication through neuronal coherence.. Trends Cog Sci.

[10] Womelsdorf T, Schoffelen JM, Oostenveld R, Singer W, Desimone R (2007). Modulation of neuronal interactions through neuronal synchornization.. Science.

[11] Supp GG, Schögl A, Trujillo-Barreto N, Müller MM, Gruber T (2007). Directed cortical information flow during human object recognition: analyzing induced EEG gamma-band responses in brain's source space.. PLoS One.

[12] Gray CM, König P, Engel AK, Singer W (1989). Oscillatory responses in cat visual cortex exhibit inter-columnar synchronization which reflects global stimulus properties.. Nature.

[13] Gray CM, Singer W (1989). Stimulus-specific neuronal oscillations in orientation columns of cat visual cortex.. Proc Natl Acad Sci U S A.

[14] Engel AK, König P, Singer W (1991). Direct physiological evidence for scene segmentation by temporal coding.. Proc Natl Acad Sci U S A.

[15] Engel AK, König P, Kreiter AK, Singer W (1991). Interhemispheric synchronization of oscillatory neuronal responses in cat visual cortex.. Science.

[16] Engel AK, Kreiter AK, König P, Singer W (1991). Synchronization of oscillatory neuronal responses between oscillatory neural responses between striate and extrastriate visual cortical areas of the cat.. Proc Natl Acad Sci U S A.

[17] Frein A, Eckhorn R, Bauer R, Woelbern T, Kehr H (1994). Stimulus specific fast oscillations at zero phase between visual areas V1 and V2of awake monkey.. Neuroreport.

[18] Goffaux V, Moraux A, Desmet S, Rossion B (2004). Human non-phase-locked gamma oscillations in experience-based perception of visual scenes.. Neurosci Lett.

[19] Rodriguez E, George N, Lachaux JP, Martinerie J, Renault B (1999). Perception's shadow: long-distance synchronization of human brain activity.. Nature.

[20] Rose M, Büchel C (2005). Neural coupling binds visual tokens to moving stimuli.. J Neurosci.

[21] Rose M, Sommer T, Büchel C (2006). Integration of local feature to a global percept by neural coupling.. Cer Cortex.

[22] Tallon-Baudry C, Bertrand O (1999). Oscillatory gamma activity in human and its role in object representation.. Trends Cog Sci.

[23] Melloni L, Molina C, Pena M, Torres D, Singer W (2007). Synchronization of neural activity across cortical areas correlates with conscious perception.. J Neurosci.

[24] Nakatani C, Ito J, Nikolaev AR, Gong P, van Leeuwen C (2005). Phase synchronization analysis of EEG during attentional blink.. J Cog Neurosci.

[25] Doesburg SM, Kitajo K, Ward LM (2005). Increased gamma-band synchrony precedes switching of conscious perceptual objects in binocular rivalry.. Neuroreport.

[26] Jensen O, Kaiser J, Lachaux JP (2007). Human gamma-frequency oscillations associated with attention and memory.. Trends Neurosci.

[27] Alkire MT, Miller J (2005). General anesthesia and the neural correlates of consciousness.. Prog Brain Res.

[28] Llinás RR, Paré D (1991). Of dreaming and wakefulness.. Neurosci.

[29] Llinás RR, Ribary U (1993). Coherent 40-Hz oscillation characterizes dream state in humans.. Proc Natl Acad Sci U S A.

[30] Llinás RR, Ribary U, Contreras D, Pedroarena (1998). The neuronal basis for consciousness.. Philos Trans R Soc Lond B Boil Sci.

[31] Canolty RT, Edwards E, Dalal SS, Soltani M, Nagarajan SS (2006). High gamma power is phase-locked to theta oscillations in human neocortex.. Science.

[32] Burgess AP, Ali L (2002). Functional connectivity of gamma EEG activity is modulated at low frequency during conscious recollection.. Int J Psychophysiol.

[33] Sauseng P, Klimesch W, Gruber WR, Birbaumer N (2008). Cross-frequency phase synchronization: a brain mechanism of memory matching and attention.. Neuroimage.

[34] Schack B, Vath N, Petsche H, Geissler HG, Moller E (2002). Phase-coupling of thet-gamma EEG rhythms during short-term memory processing.. Int J Psychophysiol.

[35] Doesburg SM, Roggeveen AB, Kitajo K, Ward LM (2008). Large-scale gamma-band phase synchronization and selective attention.. Cereb Cortex.

[36] Andrews TJ (2001). Binocular rivalry and visual awareness.. Trends Cogn Sci.

[37] Tononi G, Srinivasan R, Russell DP, Edelman GM (1998). Investigating neural correlates of conscious perception by frequency-tagged neuromagnetic responses.. Proc Natl Acad Sci U S A.

[38] Srinivasan R, Russell DP, Edelman GM, Tononi G (1999). Increased synchronization of neuromagnetic responses during conscious perception.. J Neurosci.

[39] Cosmelli D, David O, Lachaux JP, Martinerie J, Garnero (2004). Waves of consciousness: ongoing cortical patterns during binocular rivalry.. Neuroimage.

[40] Shapiro KL, Arnell KM, Raymond JE (1997). The attentional blink.. Trends Cog Sci.

[41] Fell J, Klaver P, Elger CE, Fernández G (2002). Suppression of EEG gamma activity may cause the attentional blink.. Conscious Cogn.

[42] Blake R, Fox R, McIntyre C (1971). Stochastic properties of stabilized-image binocular rivalry alternations.. J Exp Psychol.

[43] Rees G, Kreiman, Koch C (2002). Neural correlates of consciousness in humans.. Nat Rev Neurosci.

[44] Sheinberg DL, Logothetis NK (1997). The role of temporal cortical areas in perceptual organization.. Proc Natl Acad Sci U S A.

[45] Tong F, Nakayama K, Vaughan JT, Kanwisher N (1998). Binocular rivalry and visual awareness in human extrastriate cortex.. Neuron.

[46] Tong F, Meng M, Blake R (2006). Neural bases of binocular rivalry.. Trends Cog Sci.

[47] Beck D, Rees G, Frith CD, Lavine N (2002). Neural correlates of change detection and change blindness.. Nat Neurosci.

[48] Cavanna AE, Trimble MR (2006). The precuneus: a review of its functional anatomy and behavioural correlates.. Brain.

[49] Goldberg II, Harel M, Malach (2006). When the brain loses its self: prefrontal inactivation during sensorimotor processing.. Neuron.

[50] Ward LM, Algom D (1992). Mind in psychophysics.. Psychophysical Approaches to Cognition.

[51] Crick F, Koch C (1995). Are we aware of neural activity in primary visual cortex?. Nature.

[52] Blake R, Logothetis NK (2002). Visual competition.. Nat Rev Neurosci.

[53] Fries P, Roelfsema PR, Engel AK, König AK, Singer W (1997). Synchronization of oscillatory responses in visual cortex correlates with perception in interocular rivalry.. Proc Natl Acad Sci U S A.

[54] Fries P, Schröder J-H, Roelfsema PR, Singer W, Engel AK (2002). Oscillatory neuronal synchronization in primary visual cortex as a correlate of stimulus selection.. J Neurosci.

[55] Tong F (1995). Primary visual cortex and visual awareness.. Nat Rev Neurosci.

[56] Bodovitz S (2004). Consciousness is discontinuous: the perception of continuity requires conscious vectors and needs to be balanced with creativity.. Med Hypotheses.

[57] Bodovitz S (2008). Consciousness disintegrates without conscious vectors.. Med Hypotheses.

[58] Fries P (2009). Neuronal gamma-band synchronization as a fundamental process in cortical computation.. Annu Rev Neurosci.

[59] Tallon-Baudry C, Bertrand O, Peronnet F, Pernier J (1998). Induced gamma-band activity during the delay of a short-term memory task in humans.. J Neurosci.

[60] Sarnthein J, Petsche H, Rappelsberger P, Shaw GL, von Stein A (2002). Synchronization between prefrontal and posterior association cortex during human working memory.. Proc Natl Acad Sci U S A.

[61] Ward LM (2003). Synchronous neural oscillations and cognitive processes.. Trends Cogn Sci.

[62] Jensen O, Colgin LL (2007). Cross-frequency coupling between neuronal oscillations.. Trends Cogn Sci.

[63] Jung RE, Haier RJ (2007). The parieto-frontal integration (P-FIT) theory of intelligence: converging neuroimaging evidence.. Behav Brain Sci.

[64] Searle JR (1992). The rediscovery of the mind..

[65] Hebb DO (1949). The organization of behaviour: a neuropsychological theory. 378 p.

[66] Gruber T, Müller MM, Keil A (2002). Modulation of induced gamma band responses in a perceptual learning task in the human EEG.. J Cogn Neurosci.

[67] Gruber T, Maess B, Trujillo-Barreto NJ, Müller MM (2008). Sources of synchronized induced gamma-band responses during a simple object recognition task: a replication study in human MEG.. Brain Res.

[68] Ford JM, Krystal JH, Mathalon DH (2007). Neural synchrony in schizophrenia: from networks to new treatments.. Schizophr Bull.

[69] Uhlhaas PJ, Haenschel C, Nikolic D, Singer W (2008). The role of oscillations and synchrony and their putative relevance for the pathophysiology of schizophrenia.. Schizophr Bull.

[70] Lee MG, Hassani OK, Alonso A, Jones BE (2005). Cholinergic basal forebrain neurons burst with theta during waking and paradoxical sleep.. J Neurosci.

[71] Libet B, Gleason CA, Wright EW, Pearl DK (1983). Time of conscious intention to act in relation to onset of cerebral activity (readiness potential). The unconscious initiation of a freely voluntary act.. Brain.

[72] Libet B (1985). Unconscious cerebral initiative and the role of conscious will in voluntary action.. Behav Brain Sci.

[73] Dennett D, Kinsbourne M (1992). Time and the observer: the where and when of consciousness in the brain.. Behav Brain Sci.

[74] Mima T, Steger J, Schulman AE, Gelroff C, Hallet M (2000). Electroencephalographic measurement of motor cortex control of muscle activity in humans.. Clin Neurophysiol.

[75] Hoechstetter K, Bornfleth H, Weckesser D, Ille N, Berg P, Scherg M (2004). BESA source coherence: A new method to study cortical oscillatory coupling.. Brain Topogr.

[76] Green  JJ, McDonald JJ (2008). Electrical neuroimaging reveals timing of attentional control activity in human brain.. PLoS Biol.

[77] Gross J, Kujala J, Hamalainen M, Timmermann L, Schnitzler A (2001). Dynamic imaging of coherent sources: Studying neural interactions in the human brain.. Proc Natl Acad Sci U S A.

[78] Van Veen BD, van Drongelen W, Yuchtman M, Suzuki A (2007). Localization of brain electrical activity via linearly constrained minimum variance spatial filtering.. I E E E Trans Biomed Eng.

[79] Cox RW, Hyde JS (1997). Software tools for analysis and visualization of fMRI data.. NMR Biomed.

[80] Scherg M, Ille N, Bornfleth H, Berg P (2002). Advanced tools for digital EEG review: Virtual source montages, whole-head mapping, correlation, and phase analysis.. J Clin Neurophysiol.

[81] Lachaux JP, Rodriguez E, Martinerie J, Varela FJ (1999). Measuring phase synchrony in brain signals.. Hum Brain Mapp.

